# Persistence of Abdominal Pain: Did You Check for Mesenteric Vessels?

**DOI:** 10.3390/medicina59030442

**Published:** 2023-02-23

**Authors:** Jessica Piroddu, Maria Pina Dore, Giovanni Mario Pes, Pier Paolo Meloni, Giuseppe Manzoni

**Affiliations:** 1 Dipartimento di Medicina, Chirurgia e Farmacia, University of Sassari, Viale San Pietro, No. 43, 07100 Sassari, Italy; 2 Baylor College of Medicine, One Baylor Plaza, Houston, TX 77023, USA; 3 General Practitioner, Azienda Sanitaria Locale No. 1, 07100 Sassari, Italy

**Keywords:** vessel abnormalities, celiac-mesenteric trunk, aneurysms

## Abstract

The incidence of abnormalities regarding the celiac-mesenteric trunk (CMT) has been reported to be between 1% and 2.7%, whereas for visceral aneurysms the incidence is between 0.1% and 0.2% of the general population. Anatomical variations in the CMT may be the result of abnormal embryogenesis of the primitive segmental splanchnic arteries that supply the bowel and several abdominal organs. The clinical presentation may range from vague abdominal symptoms to aneurysm rupture with a significant mortality risk. In this case, we describe the clinical history of a 37-year-old man with postprandial abdominal pain likely related to the celiac-mesenteric trunk enlargement, associated with high resistance flow in the proximal site. Postprandial symptoms improved by avoiding large meals and surveillance for the CMT anomalies was recommended by cross-imaging including the echo-color-Doppler to assess blood flow modification.

## 1. Introduction

Albrecht Von Haller, a Swiss anatomist and physiologist, was the first to describe the anatomy of the celiac-mesenteric trunk (CMT) [1]. The celiac tripod, also known as the celiac trunk or celiac artery, is the first branch of the abdominal aorta arising anteriorly at the level of the vertebral bodies T12–L1. Approximately 1.5–2 cm from the aortic origin the celiac trunk continues by dividing into three major branches: (i) the common hepatic artery; (ii) the gastric artery; and (iii) the splenic artery, being the primary arterial supply of the liver, stomach, abdominal esophagus, spleen, the upper portion of the duodenum and pancreas [1].

The arrangement of the anatomical structures, and the relationship between organs, the blood, and the lymphatic vessel network are the result of the growth process, rotation, and migration during embryogenesis and fetal development [2].

Progenitor cell movement and aggregation during organogenesis are responsible for the final organs’ morphology and can be the cause of variations in arterial and venous vessels. More specifically, during embryogenesis, the main visceral arteries develop from four vascular roots derived from the primitive dorsal abdominal aorta. These four roots are joined by ventral longitudinal anastomosis. In the course of normal maturation, the gastric, hepatic, and splenic roots join to form the main celiac axis, while the fourth root develops separately into the superior mesenteric artery. An interruption of the ventral anastomosis process may lead to a wide variety of vascular anomalies [3]. Several anatomical and radiological descriptions of CMT abnormalities have been reported in the literature, including common trunks and anastomoses between the celiac trunk and the superior mesenteric artery, the inter-mesenteric arch between the superior and the inferior mesenteric arteries, or a common arterial trunk between the celiac trunk and the superior and inferior mesenteric arteries [4,5,6]. In some individuals, the celiac trunk is completely absent [4]. Therefore, anatomical variants of the celiac trunk branches may be the result of anomalous embryogenesis of the primitive segmental splanchnic arteries [7].

CMT anatomic variations are rare; they have been reported to range between 1% and 2.7% of cases [8], whereas the incidence of visceral aneurysms ranges between 0.1% and 0.2% in the general population [9]. CMT aneurysm is even rarer and occurs in only 0.25% of all visceral artery anomalies. For example, in the last 52 years, only 26 cases have been reported in the literature [10,11,12,13,14,15,16,17,18,19,20,21,22,23,24]. Depending on the location and size of the aneurysm, the mortality rate is 10–90% after rupture. The majority of visceral aneurysms occur in the splenic artery, accounting for nearly 60% of the total, while the superior mesenteric and celiac artery aneurysms account for 5% and 4%, respectively [25].

In the absence of aneurysm rupture, symptoms may be insidious and progressive with malaise, postprandial epigastric/abdominal pain or discomfort, sometimes associated with back pain, early satiety, nausea, and/or vomiting often attributed to another etiology or functional disorder, leading to delay in diagnosis [3,5,26,27].

A physical examination is usually not helpful for diagnosis. In cases where the aneurysm has expanded to a large size, it may present as a palpable mass in thin individuals [28], albeit this is uncommon. Laboratory studies are generally non-specific. Differential diagnosis may be difficult, requiring an extensive workup. The single most important step in diagnosing CMT anomalies is to suspect the disorder from the patient’s initial presentation.

Ultrasonography and cross-sectional abdominal vascular imaging, including computed tomography (CT) and magnetic resonance (MR), provide an accurate diagnosis of CMT anomalies. Echo-color-Doppler is particularly helpful for measuring blood flow inside the abnormal trunk. Moreover, imaging can simultaneously exclude additional conditions.

The suggested approach to a visceral aneurysm is early intervention. However, observation with surveillance could be an option for some small aneurysms and accordingly for trunk enlargement [29].

## 2. Case Presentation

A 37-year-old white Caucasian man came to our attention during a gastroenterological visit. Anthropometric features were: height 185 cm, weight 100 kg (body mass index 29.2 kg/m^2^). He was a former cigarette smoker and did not practice physical exercise. At the visit time, he was unemployed and consumed a balanced diet. The patient complained of gastroesophageal reflux disease (GERD) symptoms, motility-like dyspepsia, and abdominal pain localized in the epigastric region and right hypochondrium occurring nearly 30 min after meals, lasting approximately 30–60 min and exacerbated by sitting, usually improving within two hours. Additionally, the patient complained of constipation and a weight loss of 5 kg in the last two years. He had no significant comorbidities, except for appendectomy and two inguinal hernia repairs on both sides in his youth. The family history was negative for major disorders.

Physical examination did not show specific signs, although a deep palpation of the periumbilical area was able to evoke mild pain. There was no chronic therapy ongoing.

For the above-mentioned abdominal pain, the patient had undergone extensive workup over the past 2 years, including invasive and non-invasive tests suggested by different specialists, mostly surgeons. All records were carefully checked during the gastroenterological visit.

**Figure 1:**
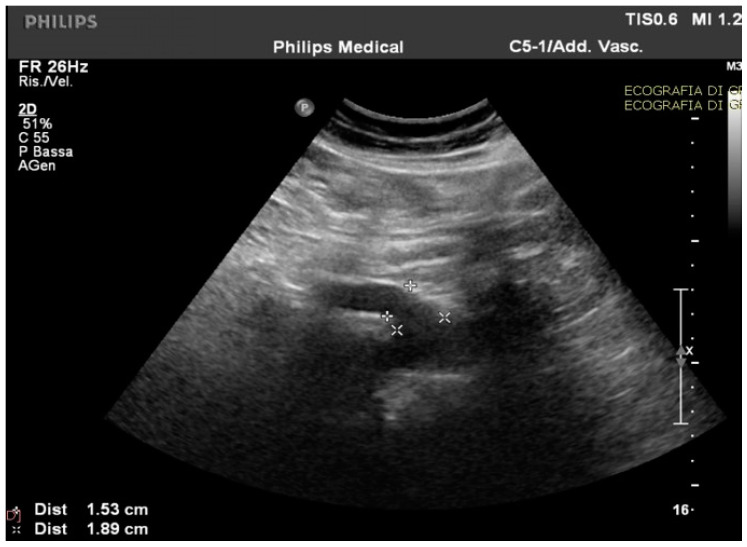
The celiac-mesenteric trunk observed by an ultrasound scan of the abdomen, indicating the size of the proximal and distal site.

Routine and specific blood and stool tests (according to the diseases’ epidemiology in our region, Sardinia, Italy) for hepatic, pancreatic, intestinal, infectious, celiac, autoimmune, and hematological diseases showed normal results. The upper endoscopy and colonoscopy were negative for significant findings. Interestingly, in the ultrasound scan of the abdomen cavity, we noticed agenesis of the left hepatic lobe, splenomegaly, and enlargement (1.89 cm at the ostium and 1.53 cm downstream) of the CMT (normally ranging between 0.7 to 1 cm) (Figure 1).

**Figure 2:**
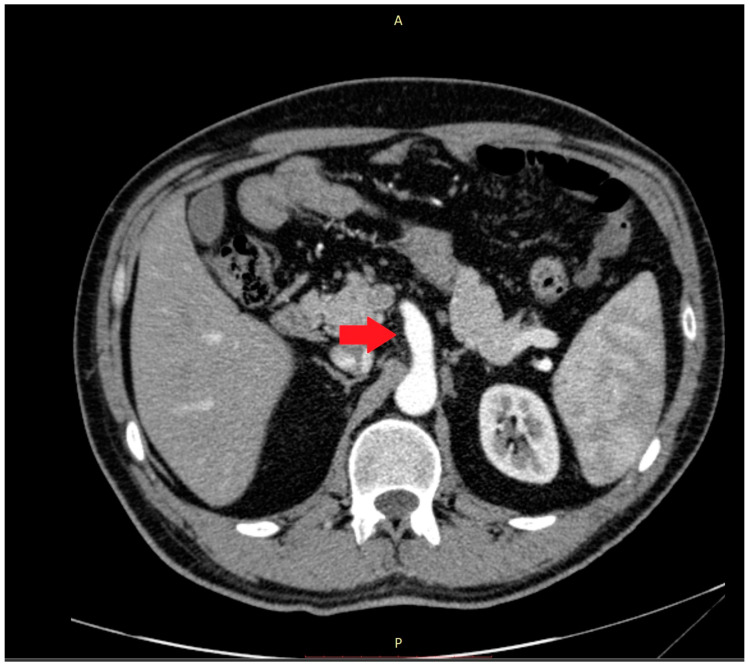
The computer tomography scan confirmed the agenesis of the left hepatic lobe, splenomegaly, and an enlarged celiac-mesenteric trunk (red arrow).

**Figure 3:**
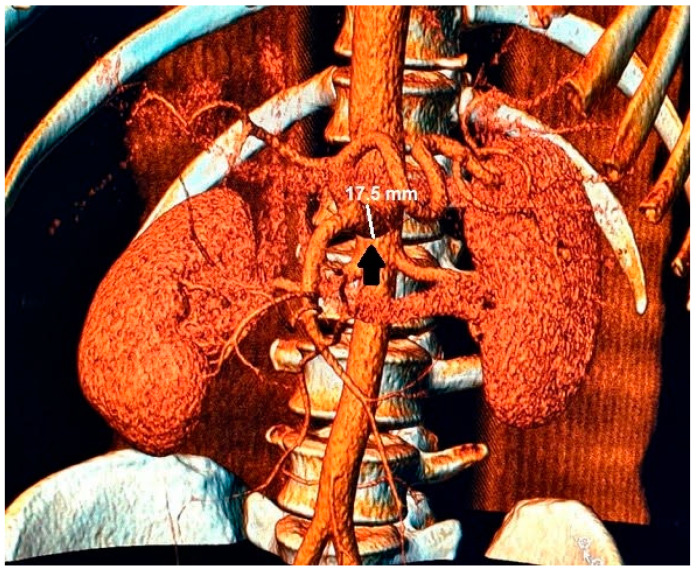
In the 3D reconstruction of CT scan images, the enlarged celiac-mesenteric trunk can be observed indicated by a black arrow.

Abnormalities were also present in the CT scan with and without contrast medium (Figure 2 and Figure 3).

**Figure 4:**
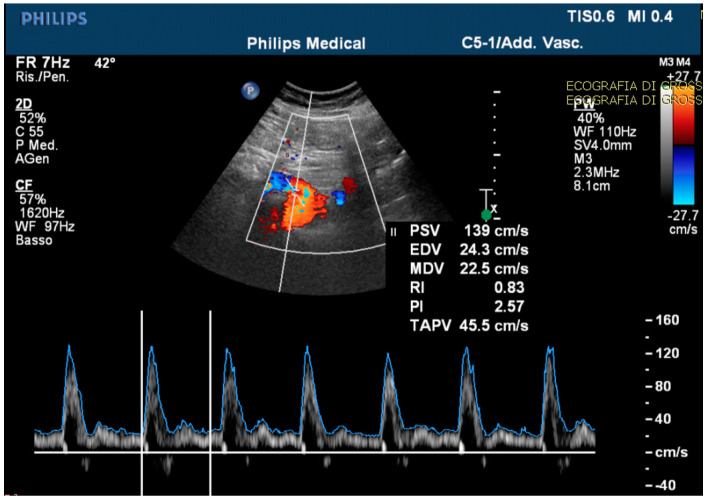
The echo-color-Doppler detected a high resistance flow in the proximal site of the celiac-mesenteric trunk, usually ranging from systolic velocity peaks between 90–100 cm/s (PSV); 30–65 cm/s end diastolic velocity peaks (EDV); and a pulsatility index (PI) of 1.5 ± 0.02.

The echo-color-Doppler revealed a high resistance flow in the proximal site of the CMT (Figure 4).

By comparing previous and current imaging tests, an increase of 5 mm in CMT diameter in 5 years was observed.

The consulted team of vascular surgeons recommended surveillance over intervention for the CMT anomalies by cross-imaging, according to the guidelines of the European Society of Vascular Surgery [30].

High doses of second-generation proton pump inhibitors twice daily, in addition to prokinetics, were prescribed, and lifestyle with dietary modification was proposed. In the follow-up visit (three weeks later), the patient reported an improvement in GERD and motility-like dyspepsia symptoms, despite the persistence of the post-prandial pain exacerbated by large meals. Because of this, the patient was asked to avoid large meals with high fat content. More specifically, the patient was advised to reduce the main meal portion sizes (lunch and dinner) and, in case of hunger, to add snacks between meals. At the third follow-up visit (2 months later), the patient reported an improvement in abdominal symptoms and quality of life through the adoption of a different eating pattern. Moreover, he maintained a steady weight.

## 3. Discussion

Causes of chronic abdominal pain and weight loss in adults are several and frequently prompt an extensive workup. More specifically, our patient complained of upper abdominal pain located in the right upper quadrant and epigastric region, characteristic locations for biliary and/or liver etiologies. However, laboratory studies were normal over time, excluding hepatobiliary disorders. Due to their rarity, visceral artery anomalies and associated modifications in blood flow are often unsuspected in patients reporting abdominal complaints. The majority of visceral abnormalities and/or aneurysms are asymptomatic and detected during autopsy. In our case, the CMT anomaly was labeled by the radiologist as an enlargement. Although there are no specific symptoms reported in the literature for CMT anomalies, in the case of a hepatic aneurysm, symptoms are represented by nausea and pain in the right hypochondrium or mesoepigastrium radiating to the back. Patients with splenic artery aneurysms complain of nausea and vague abdominal discomfort in the mesoepigastric quadrant or left hypochondrium, associated sometimes with left shoulder discomfort due to diaphragm irritation. Almost half of patients with splenic artery aneurysms present with moderate splenomegaly [26]. Most celiac artery aneurysms are asymptomatic and rarely associated with mesoepigastrium pain radiating to the back, mimicking the symptoms of pancreatitis [27]. Symptoms related to an aneurysm of the superior mesenteric artery are generally nonspecific, but if there is an aneurysm-related thrombus, ischemic symptoms may occur, resulting in pain after meals [5]. Similarly, to angina abdominis, our patient also complained of abdominal pain after meals that almost completely resolved after changing eating pattern, although a different cause of the pain could not be ruled out.

## 4. Conclusions

This case of unexplained abdominal pain includes a difficult-to-diagnose condition that is not frequently encountered by most clinicians but is nonetheless important to accurately recognize. The clinical presentation of CTM anomalies may range from vague abdominal symptoms to aneurysm rupture with a significant mortality risk especially when complicated by a high blood flow resistance. Postprandial symptoms improved by avoiding large meals and surveillance was recommended by cross-imaging, including echo-color-Doppler, to assess the magnitude of blood flow modification.

## Data Availability

Data and material are available on reasonable request.
